# Antiradical and Antioxidative Activity of Azocalix[4]arene Derivatives: Combined Experimental and Theoretical Study

**DOI:** 10.3390/molecules24030485

**Published:** 2019-01-29

**Authors:** Jiaqi Ni, Lilin Lu, Yi Liu

**Affiliations:** 1 State Key Laboratory of Refractories and Metallurgy, Wuhan University of Science and Technology, Wuhan 430081, China; 15671569690n@sina.com; 2 Hubei Province Key Laboratory of Coal Conversion and New Carbon Materials, School of Chemistry and Chemical Engineering, Wuhan University of Science and Technology, Wuhan 430081, China; yiliuchem@whu.edu.cn; 3 State Key Laboratory of Virology & Key Laboratory of Analytical Chemistry for Biology and Medicine, College of Chemistry and Molecular Science, Wuhan University, Wuhan 430072, China

**Keywords:** azocalix[4]arene, antiradical, antioxidant, mechanism, density functional theory

## Abstract

Developing antioxidants with high efficiency is fundamentally important for the protection of living cells and engineering materials against oxidative damage. In this present study, two azocalix[4]arene derivatives were synthesized via a diazo coupling reaction between calix[4]arene and diazonium salts. Their antiradical and antioxidative performances were evaluated by hydroxyl radical scavenging and pyrogallol autoxidation inhibition experiments. Combined with theoretical studies, the antiradical and antioxidative mechanisms have been explored. The results demonstrated that these two azocalix[4]arene derivatives both exhibited remarkable antiradical and antioxidative activity. The macrocyclic framework of the calix[4]arene and para-azo substituent group at the upper rim of calix[4]arene contributed synergistically and importantly to its excellent antiradical and antioxidant activity.

## 1. Introduction

Reactive oxygen species (ROS) are widely found in a number of life-sustaining biochemical processes in living cells, such as aerobic metabolism [1]. Furthermore, these types of reactive species are involved in many material fields (e.g., polymers and lubricants) and industries (e.g., food industry). Oxidative degradation resulting from reactive species is a general phenomenon that occurs in living cells and engineering materials, leading to many severe problems, such as cardiovascular disease, cancer, chronic inflammatory diseases [2], and a sharp deterioration of the mechanical properties of engineering materials. Therefore, developing efficient antioxidants to scavenge reactive oxygen species and to alleviate oxidative damage has always been an imperative research issue for many decades.

Calixarene, which are the cyclic oligomers obtained by the condensation of formaldehyde with p-alkylphenols, has been considered to be the third generation supramolecular host molecule after crown ethers and cyclodextrins. It has been extensively used in molecular recognition, ion sensing and drug delivery [3,4,5]. Due to the hindered phenol molecular structure, calixarene and its derivatives were also reported to be part of a class of excellent antioxidants with inherent antioxidative activities. *p*-tertbutyl-calix[4]arene was reported to be effective light and heat stabilizers for polyolefins [6,7]. The radiation stability of polypropylene can be significantly improved by *p*-tert-butylcalix[n]arene [8]. Low density polyethylenes and high density polyethylenes could be effectively stabilized by *p*-tertbutyl-calix[4]arene and *p*-tertbutyl-calix[6]arene, respectively [9]. Calix[n]arenes that feature sulphonate groups above and/or below the plane of the macrocycle have intrinsic antioxidant capacity and antibacterial activity [10]. Four dihydropyrimidine moieties were grafted at the upper rim of calix[4]arene, which produced calix[4]arene-based dihydropyrimidines that possess amplified antiradical activity in comparison to the corresponding monomers [11]. Furthermore, a calix[4]arene-like tetramer of resveratrol was reported to exhibit significantly improved antioxidative activity with respect to resveratrol [12]. Calixarene-based antioxidant C-methylcalix[4]resorcinarene also exhibited superior antioxidative efficiency in natural rubber vulcanizates compared to that of classical antioxidant 2,6-di-tert-butyl-4-methylphenol (BHT) [13]. In addition, calix[4]arene has also been used for rigid molecular scaffolds to cluster hydroxycinnamic acid in order to improve radical scavenging and antioxidative activity [14].

**Figure 1:**
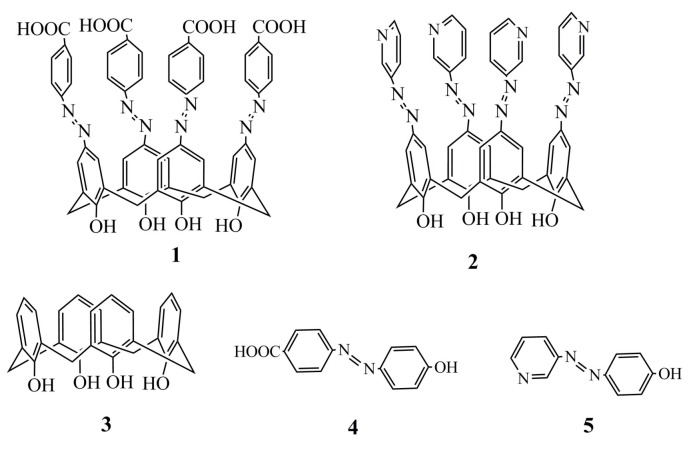
Chemical structures of azocalix[4]arene derivatives **1** and **2**, calix[4]arene (**3**), *p*-carboxyphenyl-azo-phenol (**4**) and 3-aminopyridine-azo-phenol (**5**).

**Figure 2:**
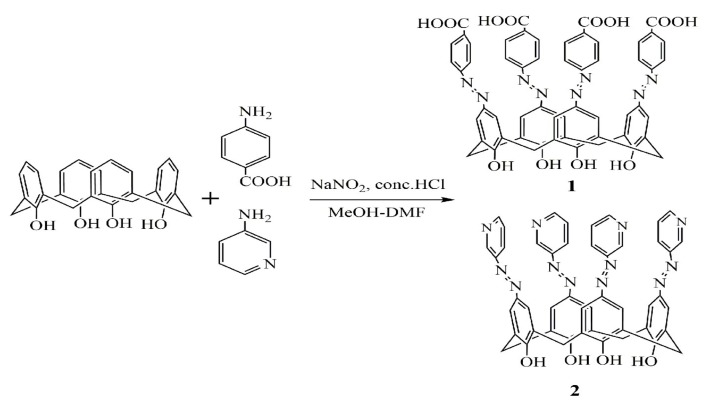
Synthesis route of azocalix[4]arene derivatives **1** and **2**.

In this work, two azocalix[4]arene derivatives (Figure 1), which were namely 5,11,17,23-tetrakis[(p-carboxyphenyl)azo]-25,26,27,28-tetrahydroxycalix[4]arene (**1**) and 5,11,17,23-tetrakis[(3-pyridine)azo]-25,26,27,28-tetra hydroxycalix[4]arene (**2**), were synthesized via a diazo coupling reaction between calix[4]arene and diazonium salts. It was then characterized by ^1^H-NMR and IR (Figure 2). Their antiradical capacities were evaluated by hydroxyl radical (·OH) scavenging experiments and their antioxidative activities were tested by a pyrogallol autoxidation inhibiting experiment. To elucidate the antiradical and antioxidative mechanisms, combined experimental and theoretical studies were also performed to explore the necessary pharmacophores that are responsible for their antiradical and antioxidative activities. The effect of the calix[4]arene framework and azo substituent group at the upper rim of calix[4]arene on the antiradical and antioxidative activities were experimentally investigated by comparison with calix[4]arene (**3**) and the corresponding monomer model compounds, *p*-carboxyphenyl-azo-phenol (**4**) and 3-aminopyridine-azo-phenol (**5**). The antiradical and antioxidative mechanisms were theoretically studied on the basis of hydrogen abstraction transfer (HAT) and sequential proton loss electron transfer (SPLET) pathways.

## 2. Results and Discussion

### 2.1. Hydroxyl Radical Scavenging Activity

In this work, hydroxyl radicals (·OH) were produced via irradiation decomposition of H_2_O_2_ [15,16] before being reacted with terephthalic acid to form a fluorescent compound hydroxyterephthalic acid (HTA, λ_em_ 425 nm) [17,18]. The reactions are described as follows:

(1) $$H_{2}O_{2}\overset{\;254\;\mathrm{nm}\;\mathrm{UV}\;\mathrm{irradiation}\;}{\to }2•\mathrm{OH}$$

(2) •OH+terephthalic acid (TA)→HTA(fluorescent)

**Figure 3:**
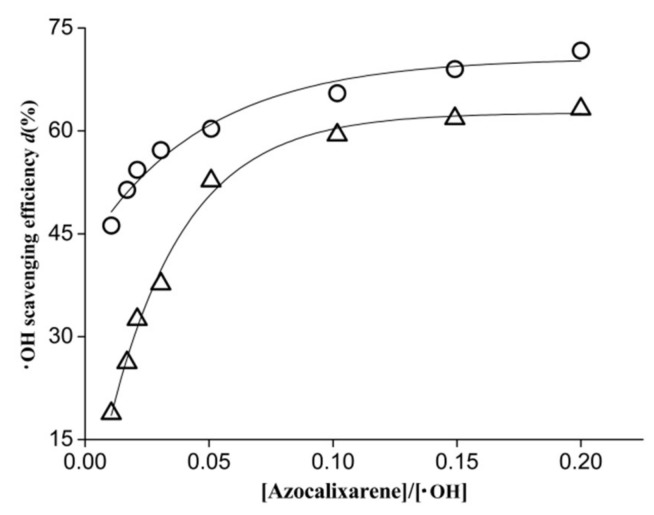
Hydroxyl radical scavenging efficiency as a function of the concentration ratio of [azocalixarene]/[·OH] where cycle (○) and triangle (△) represent azocalix[4]arene derivatives 1 and 2, respectively.

In the antiradical experiment, the antioxidant competes with terephthalic acid to trap hydroxyl radicals, thus inhibiting the production of HTA and leading to a decrease in fluorescence intensity. The results of hydroxyl radical scavenging of azocalixarene derivatives **1** and **2** are shown in Figure 3. As shown in Figure 3, a significant decrease in fluorescence intensity at 425 nm was induced by azocalixarene derivatives **1** and **2**. The hydroxyl radical scavenging efficiency was calculated according to the following equation:

*d* = (I_0_ − I_f_)/I_0_ × 100% (3)

where the I_0_ and I_f_ represent the fluorescence intensity before and after the addition of azocalixarene, respectively.

Figure 3 shows the plot of hydroxyl radical scavenging efficiency (*d*) against the concentration ratio of [azocalixarene]/[·OH]. At the [azocalixarene]/[·OH] ratio of 0.02, the hydroxyl radical scavenging efficiency of **1** and **2** are approximately 54.3% and 32.5%, respectively. When the concentration ratio increased to approximately 0.2, the hydroxyl radical scavenging efficiencies increased to 71.7% and 63.2%, respectively. Compared with compound **2**, the calix[4]arene derivative **1** showed higher hydroxyl radical scavenging activity, especially at a low concentration range. For instance, at the [azocalixarene]/[·OH] ratio of 0.01, the hydroxyl radical scavenging efficiency of **1** is about 45%, which is almost three-fold of that (approximately 15%.) of **2**, demonstrating that compound **1** possesses greater antiradical capacity than compound **2**. These results demonstrated that the two investigated azocalixarene derivatives both exhibited significant scavenging activity for hydroxyl radicals.

### 2.2. Pyrogallol Autoxidation Inhibition Activity

**Figure 4:**
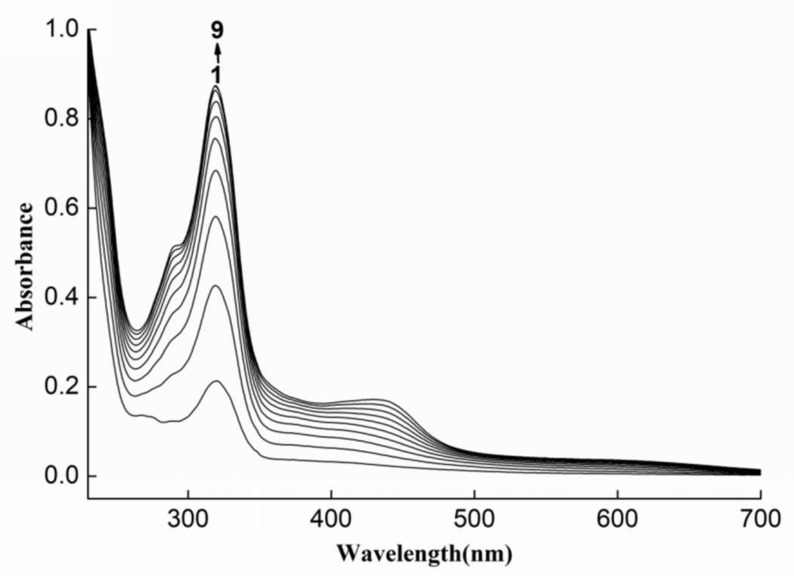
Absorption spectrum of pyrogallol in Tris-HCl buffer solution (pH = 8.2) after autoxidation for 0.5, 4.0, 8.0, 12.0, 16.0, 20.0, 24.0, 28.0 and 32.0 min (1–9).

The autoxidation of pyrogallol in an alkaline aqueous solution has been extensively observed in previous works [19,20]. In this present study, pyrogallol autoxidation experiments have also been performed and the UV–vis absorption spectrum was constantly measured (displayed in Figure 4). An increasing absorption peak at 320 nm was observed and the absorbance reached its maximum after pyrogallol autoxidation for 30 min. In this work, the pyrogallol autoxidation inhibition experiments of azocalixarene derivatives were allowed to proceed for 30 min before the absorbance at 320 nm was measured to calculate the inhibition efficiency *E*(%) according to the following equation:

(4) $$E\%=\frac{A_{0}-A}{A_{0}}\times 100\%$$

where *A*_0_ was the absorbance at 320 nm after autoxidation for 30 min when no antioxidant was added and *A* was the absorbance after azocalixarene was added as an antioxidant.

**Figure 5:**
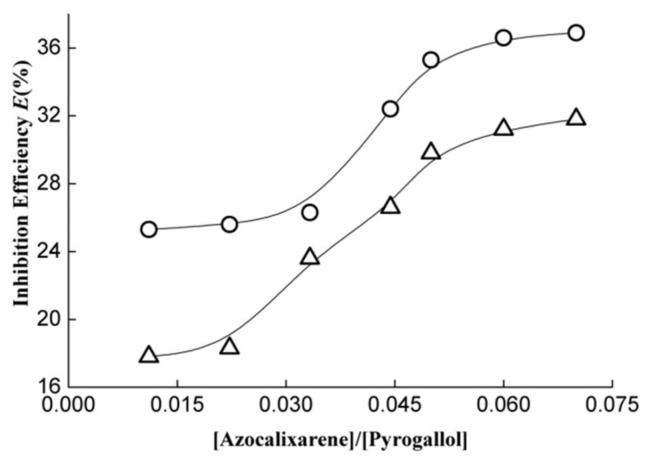
Pyrogallol autoxidation inhibition efficiency as a function of the concentration ratio of [azocalixarene]/[pyrogallol] where cycle (○) and triangle (△) represent azocalix[4]arene derivatives 1 and 2, respectively.

The performances of azocalixarene derivatives in the pyrogallol autoxidation inhibition experiment are displayed in Figure 5. As shown in Figure 5, the investigated azocalix[4]arene derivatives **1** and **2** both exhibited remarkable activity in pyrogallol autoxidation inhibition. When [azocalixarene]/[pyrogallol] ratio was equal to 0.01, the inhibition efficiencies reach about 25% and 18% for **1** and **2**, respectively. With an increase in the concentration ratio of [azocalixarene]/[pyrogallol], the inhibition efficiency increases correspondingly. When the concentration ratio increased to 0.07, the inhibition efficiency increased to 36.9% and 31.8%, respectively, for **1** and **2**. Similarly, compound **1** exhibited higher activity in the inhibition of pyrogallol autoxidation compared to compound **2** over the whole testing range of [azocalixarene]/[pyrogallol] ratios, indicating that the azocalixarene derivatives **1** possess more excellent antioxidative capacity.

The performance of calix[4]arene (**3**), *p*-carboxyphenyl-azo-phenol (**4**) and 3-aminopyridine-azo-phenol (**5**) in hydroxyl radical scavenging and pyrogallol autoxidation inhibition experiments were also investigated and compared to each other. An [antioxidant]/[·OH] ratio of 0.1 and [antioxidant]/[pyrogallol] of 0.05 were selected, respectively, for hydroxyl radical scavenging and pyrogallol autoxidation inhibition experiments under identical experimental conditions. The results manifested that calix[4]arene, *p*-carboxyphenyl-azo-phenol and 3-aminopyridine-azo-phenol exhibited negligible antiradical capacity and antioxidative activity. The hydroxyl radical scavenging efficiencies of calix[4]arene, *p*-carboxyphenyl-azo-phenol and 3-aminopyridine-azo-phenol were 2.8%, 5.2% and 3.6%, respectively. In pyrogallol autoxidation inhibition experiments, the inhibition efficiencies of these compounds were 1.9%, 1.6% and 1.4%, respectively. These results demonstrated that the macrocyclic framework of calix[4]arene or azo group was not the crucial factor that is individually responsible for determining the capacity of azocalix[4]arene derivatives. It was the synergistic effect of the macrocyclic framework of calix[4]arene and azo group at the upper rim of calix[4]arene that might result in the significant antiradical and antioxidative activity of azocalixarene derivatives **1** and **2**.

### 2.3. Phenolic O–H BDEs, Proton Affinities (PAs) and Electron Transfer Enthalpies of Phenolate Anions

For phenolic antioxidants, their radical scavenging and antioxidative activity rely significantly on their ability to transfer their phenolic H-atom to radical species in order to break the chain reaction. Generally, there are two pathways for H-atom transfer, which are namely the hydrogen abstraction transfer (HAT) and sequential proton loss electron transfer (SPLET) [21,22]. In the former case, the antioxidant donates a H-atom via the homolytic dissociation of the phenolic O–H bond, which is under the control of the O–H bond dissociation enthalpy (BDE). The lower O–H BDE has a weaker phenolic O–H bond and favors the O–H homolytic dissociation donating the H-atom. In the latter case, the H-atom transfer process is governed by the acidity of phenolic O–H, which is generally characterized by the proton affinity (PA) of the corresponding phenolate anion. A lower PA means the stronger O–H acidity, which is more favorable for the heterolytic dissociation. Compared with a neutral molecule, the produced phenolate anion has the lower ionization potential, which facilitates the sequential electron transfer process to produce the phenoxyl radical.

Considerable efforts have been devoted to the measurement of O–H BDE using versatile techniques, such as photoacoustic calorimetry [23] and EPR spectroscopy [24,25]. Quantum mechanical methods have also been extensively used for evaluating the O–H BDE [26,27]. In this present work, DFT calculations have been performed at the BP86/6-311+G(d,p) level of theory to calculate the O–H BDEs and the PAs of phenolate anions of azocalixarene derivatives **1** and **2** in an aqueous solution in order to explore the antiradical and antioxidative mechanisms [12,28]. For comparison, the O–H BDEs of calix[4]arene, *p*-carboxyphenyl-azo-phenol and 3-aminopyridine-azo-phenol and the PAs of phenolate anions of them are also calculated. All of the results are listed in Table 1.

As shown in Table 1, the calculated O–H BDE of calix[4]arene, *p*-carboxyphenyl-azo-phenol and 3-aminopyridine-azo-phenol is 82.17, 81.26 and 81.42 kcal/mol, respectively. In azocalix[4]arene derivatives **1** and **2**, the O–H BDEs were 76.39 and 77.97 kcal/mol, respectively. They are distinctly lower than those of calix[4]arene, *p*-carboxyphenyl-azo-phenol and 3-aminopyridine-azo-phenol. This indicated that the phenolic O–H bonds in azocalix[4]arene derivatives **1** and **2** were significantly weakened, thus facilitating H-atom being donated from the phenolic O–H group to break the chain reaction of radicals. This result is consistent with the above-mentioned experimental findings that azocalix[4]arene derivatives showed superior hydroxyl radical scavenging capacity compared to calix[4]arene, *p*-carboxyphenyl-azo-phenol and 3-aminopyridine-azo-phenol.

The proton affinities and electron transfer enthalpy of phenolate anions were also investigated to explore the acidity of phenolic O–H and electron loss capacity of phenolate anions. For phenolate anions of all investigated compounds, the calculated electron transfer enthalpy is significantly lower than the proton affinity, indicating that the first proton dissociation is the rate-determined step of the sequential proton loss electron transfer (SPLET) process. For calix[4]arene, *p*-carboxyphenyl-azo-phenol and 3-aminopyridine-azo-phenol, their proton affinities were calculated to be 276.59, 278.57 and 279.54 kcal/mol, respectively. The calculated proton affinities of azocalix[4]arene derivatives **1** and **2** are 265.85 kcal/mol and 266.70 kcal/mol, respectively. The distinct decrease in the proton affinity of azocalix[4]arene derivatives with respect to those of calix[4]arene, *p*-carboxyphenyl-azo-phenol and 3-aminopyridine-azo-phenol indicated the stronger acidity of the O–H group, which would facilitate the production of phenolate anions.

As concluded from the above-mentioned results, the synergistic effect of macrocyclic framework of calix[4]arene and azo group at the upper rim of calix[4]arene is responsible for the lower O–H BDEs and proton affinity of the phenolate anion, thus resulting in the excellent radical scavenging activity and pyrogallol autoxidation inhibition ability of azocalix[4]arene derivatives **1** and **2**.

In previous works, the superoxide anion radical (·O_2_^−^) has been confirmed to be involved in the autoxidation of pyrogallol [29] and inhibition of pyrogallol autoxidation was widely used to measure the capacity of antioxidants to scavenge superoxide anion radicals [20]. In this work, the investigated azocalixarene derivatives exhibited excellent activity for pyrogallol autoxidation inhibition, which might be explained by the strong acidity of their O–H group. Firstly, the negatively charged superoxide anion radical, which was produced during pyrogallol autoxidation, accepted the proton donated by azocalix[4]arene derivatives to form a neutral radical ·O_2_H. Secondly, the electron was transferred from the phenolate anion of the azocalixarene derivative to ·O_2_H and subsequently quenched the anion. As shown by the relatively higher PA of phenolate anion with respect to O–H BDE in Table 1, the investigated azocalix[4]arene derivatives possess more excellent activity in hydroxyl radical scavenging than in superoxide anion radical scavenging, thus exerting higher efficiency in the hydroxyl radical scavenging experiment but relatively lower efficiency in the pyrogallol autoxidation inhibition experiment. This is consistent with the experimental performance as the highest efficiency of hydroxyl radical scavenging for the investigated azocalix[4]arene derivatives was about 71.7%, whereas the highest efficiency of pyrogallol autoxidation inhibition was only about 36.9%.

### 2.4. Spin Density Distribution Analysis of Phenoxyl Radicals

**Figure 6:**
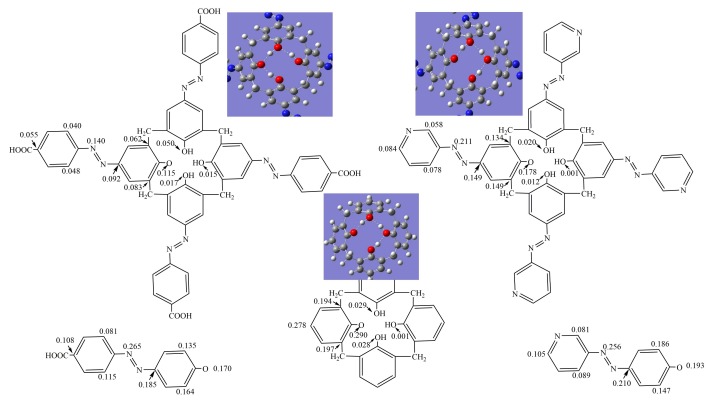
Spin density distribution in the phenoxyl radicals of azocalix[4]arene derivatives **1** and **2**, calix[4]arene, *p*-carboxyphenyl-azo-phenol and 3-aminopyridine-azo-phenol.

The stability of the phenoxyl radical produced via the HAT or SPLET process is another important index for antioxidants. It is desirable that a phenoxyl radical is produced with enough stability because otherwise it may not be able to easily induce a new chain reaction between itself and substrates. The unpaired electron distribution in phenoxyl radical plays an important role in determining its stability. More delocalized unpaired electrons in the radical creates more stability in the radical. In this present study, a spin density distribution analysis was performed and displayed in Figure 6 to explore the unpaired electron delocalization in the phenoxyl radical of azocalix[4]arene derivatives. Furthermore, the phenoxyl radicals of calix[4]arene, *p*-carboxyphenyl-azo-phenol and 3-aminopyridine-azo-phenol were also investigated and compared.

As shown in Figure 6, in the phenoxyl radicals of *p*-carboxyphenyl-azo-phenol and 3-aminopyridine-azo-phenol, the unpaired electron is mainly confined to the remaining phenolic oxygen atom, carbon atom in benzene ring and the nitrogen atom in the azo group. The spin density on the phenolic O-atom are 0.170 and 0.193, respectively. In the phenoxyl radical of calix[4]arene, the unpaired electron was confined to the remaining phenolic O-atom and the connected phenol ring, which results in the spin density of 0.290 on the remaining phenolic O-atom. In phenoxyl radicals of azocalix[4]arene derivatives **1** and **2**, the spin density on the remaining phenolic O-atom decreased to 0.115 and 0.178, respectively. These values are significantly lower than the corresponding spin density in the phenoxyl radicals of calix[4]arene, *p*-carboxyphenyl-azo-phenol and 3-aminopyridine-azo-phenol. These results demonstrated that the unpaired electron in phenoxyl radicals is more delocalized while the macrocyclic framework of calix[4]arene and azo substituent group at the upper rim of calixarene contributed synergistically and importantly to the delocalization of unpaired electrons.

## 3. Materials and Methods

### 3.1. Synthesis of 5,11,17,23-tetrakis[(p-carboxyphenyl)azo]-25,26,27,28-tetrahydroxycalix[4]arene (**1**) and 5,11,17,23-tetrakis[(3-pyridine) azo]-25,26,27,28-tetrahydroxycalix[4]arene (**2**)

*p*-tert-butylcalix[4]arene was synthesized according to the method described in literature [30]. Calix[4]arene was prepared by the debutylation of *p*-tertbutylcalix[4]arene [31]. The azocalix[4]arene derivatives (**1** and **2**) were synthesized via a diazo coupling reaction between calix[4]arene and diazonium salts [32,33,34].

In a typical synthesis, 0.685 g of *p*-aminobenzoic acid crystal was added to 10 mL of water while constantly stirring and heating. After it was dissolved, the solution was cooled down to 5 °C in an ice bath before a solution of 0.345 g NaNO_2_ dissolved in 2.5 mL of water was added. The solution was acidified by a diluted aqueous HCl solution. After half an hour of stirring, approximately 0.2 g of urea was added and the solution was stirred for a further 10 min to obtain the solution of diazonium salt. The diazonium salt solution was added into a cold solution of 1.0 g (2.36 mmol) calix[4]arene and 2.46 g (30 mmol) of anhydrous sodium acetate in 26 mL of MeOH-DMF (5:8 *v*/*v*) in order to produce a red suspension. This red reactant was allowed to couple for two hours in an ice bath, which was subsequently acidified by 150 mL of a HCl aqueous solution and warmed at 60 °C for 30 min to produce a reddish viscous solid. After being filtered out, the solid was washed with water and methanol several times before being dried under vacuum to obtain 5,11,17,23-tetrakis[(*p*-carboxyphenyl)azo]-25,26,27,28-tetrahydroxy-calix[4]arene (**1**). We obtained the following results. ^1^H-NMR(DMSO-D), δ(ppm): 3.8 and 4.0 (d, 8H,ArCH_2_Ar), 8.0~8.1(m, 4H, ArOH), 7.1~7.8(m, 24H, Ar), 8~14(s, 4H, COOH). IR, ν: 3421 cm^−1^ and 3190 cm^−1^ (OH), 1602 cm^−1^ (N=N), 1408 cm^−1^ (C=O/ArCOOH). In a similar procedure, 0.77 g (10 mmol) 3-aminopyridine was used to synthesize 5,11,17,23-tetrakis[(3-pyridine) azo]-25,26,27,28-tetrahydroxycalix[4]arene (2). ^1^H-NMR(MeOD), δ(ppm): 4.6 and 4.9 (d, 8H,ArCH_2_Ar), 8.6 (s, 4H, ArOH), 6.8~8.1 (m, 24H, Ar). IR, ν: 3176 cm^−1^ (OH), 1594 cm^−1^ (N=N), 1676cm^−1^ (Ar), 1649cm^−1^(pyridine). For comparison, *p*-carboxyphenyl-azo-phenol and 3-aminopyridine-azo-phenol were also synthesized in a similar way via the diazo coupling reaction between phenol and diazonium salts. All chemicals and solvents used in this work were of analytical grade and used without further purification unless otherwise mentioned. Furthermore, doubly distilled water was used for the solution preparation.

### 3.2. Hydroxyl Radical Scavenging and Pyrogallol Autoxidation Inhibition

Hydroxyl radicals were produced via the irradiation decomposition of H_2_O_2_ under 254-nm UV light. The amount of hydroxyl radicals was determined on the basis of the fluorescence intensity of hydroxyterephthalic acid (HTA) [35]. The details of the procedure are provided as follows. A total of 100 μL of H_2_O_2_ solution (20 mM), a certain volume of azocalixarene derivatives solution (0.02 mM) and 100 μL of terephthalic acid (TA) solution (2 mM) were mixed before the final volume of mixed solution was adjusted to 5 mL. After stirring for thirty seconds, the solution was transferred into a 1-cm quartz cell and irradiated directly under 254-nm UV light for 20 min at room temperature and the fluorescence intensity (λ_ex_ 312 nm) at 425 nm was measured immediately. The pyrogallol autoxidation inhibition performances of the investigated samples were tested according to the following procedure. We mixed 0.3 mL of a pyrogallol aqueous solution (3 mM), 4.5 mL of a Tris-HCl buffer solution (50 mM, pH = 8.2) and a certain volume of azocalixarene derivatives solution (0.02mM) before the final volume was adjusted to 9 mL. After being kept in a water bath at 25 °C for 30 min, the absorbance at 320 nm was measured. In control experiments, the azocalixarene derivatives have been replaced by other compounds, such as calix[4]arene, *p*-carboxyphenyl-azophenol and 3-pyridine-azophenol.

### 3.3. Density Functional Theory Study of the Antiradical and Antioxidative Mechanism

Geometries of azocalixarene derivatives (**1** and **2**), related phenoxyl radicals and phenolate anions were optimized by employing a generalized gradient approximation using the BP86 functional [36,37] and 6-31G(d) basis set. Unrestricted formulation was applied for open-shell radical species. The energies of all investigated chemical systems were refined by performing single-point calculations at BP86/6-311+G(d,p) level of theory in an aqueous solution. A polarizable continuum model (PCM) was used to simulate the aqueous solution environment with the dielectric constant of 78.3553. The antioxidative and antiradical activities of azocalixarene derivatives were theoretically studied based on the hydrogen abstraction transfer (HAT) mechanism and sequential proton loss electron transfer (SPLET) mechanism. Zero-point corrected energies and enthalpies were selected to calculate the O–H bond dissociation enthalpy (BDE), the proton affinity (PA) and electron transfer enthalpy (ETE) of phenolate anions according to the method described in previous works [21,22]. All computations in this work are performed with the Gaussian 09W suite of programs (Gaussian, Inc., Pittsburgh, PA, USA) [38].

## 4. Conclusions

In conclusion, azocalix[4]arene derivatives **1** and **2** have been synthesized via the diazo coupling reaction between calix[4]arene and diazonium salts. Hydroxyl radical scavenging and pyrogallol autoxidation inhibition experiments demonstrated that the two investigated azocalix[4]arene derivatives both exhibited remarkable antiradical and antioxidative activity. The compound **1** showed higher activity in hydroxyl radical scavenging and pyrogallol autoxidation inhibition, with the highest hydroxyl radical scavenging efficiency and pyrogallol autoxidation inhibition efficiency found to be about 71.7% and 36.9%, respectively. Combined experimental and theoretical studies revealed that the macrocyclic framework of calix[4]arene and *para*-azo substituent group at the upper rim of calix[4]arene mainly contributed to their antiradical and antioxidant activity due to the poor antiradical and antioxidative performance of calix[4]arene, *p*-carboxyphenyl-azo-phenol and 3-aminopyridine-azo-phenol. DFT calculations at BP86/6-311+G(d,p) level of theory demonstrated that phenolic O–H BDEs and proton affinities of phenolate anions of azocalix[4]arene derivatives **1** and **2** were significantly lower than that in calix[4]arene, *p*-carboxyphenyl-azo-phenol and 3-aminopyridine-azo-phenol. Spin density analysis revealed that the spin density on the remaining phenolic O-atom in the phenoxyl radicals of azocalix[4]arene derivatives **1** and **2** was distinctly lower than those in calix[4]arene, *p*-carboxyphenyl-azo-phenol and 3-aminopyridine-azo-phenol. This was indicative of a higher degree of unpaired electron delocalization and more stable phenoxyl radicals in the phenoxyl radicals of azocalix[4]arene derivatives **1** and **2**. These results confirmed that the synergetic effect of macrocyclic framework of calix[4]arene and *para*-azo substituent group at the upper rim of calix[4]arene was responsible for the excellent antiradical and antioxidative activity of the investigated azocalix[4]arene derivatives. Thus, these derivatives would be a promising antioxidant candidate to protect living cells and engineering materials against radical and oxidative damage.

## Figures and Tables

**Table 1:** The calculated phenolic O–H bond dissociation enthalpies (BDEs) and proton affinities (PAs) of phenolate anions of investigated chemical systems at BP86/6-311+G(d,p) level of theory.

Compounds	O–H BDEs (kcal/mol)	Phenolate Anion	
		PAs (kcal/mol)	ETEs (kcal/mol)
Calix[4]arene	82.17 *	276.59	117.74
*p*-carboxyphenyl-azo-phenol	81.26	278.57	115.21
3-aminopyridine-azo-phenol	81.42	279.54	114.41
**1**	76.39 *	265.85	122.89
**2**	77.97 *	266.70	123.64

* For calix[4]arene compounds, only one phenolic hydroxyl was arbitrarily selected to be studied by DFT calculations.
